# Preserving Fertility in Children and Adolescents with Cancer

**DOI:** 10.3390/children1020166

**Published:** 2014-08-26

**Authors:** Jennifer M. Levine

**Affiliations:** Division of Pediatric Hematology, Oncology and Stem Cell Transplant, Columbia University Medical Center, 161 Fort Washington Avenue, IP-7, New York, NY 10032, USA; E-Mail: jl175@columbia.edu; Tel.: +212-305-5808; Fax: +212-305-5848

**Keywords:** pediatric cancer, survivorship, late effects, fertility

## Abstract

In the face of excellent survival rates for pediatric and adolescent cancer, preserving the opportunity to have biological children is an important component of long term quality of life. Yet, modern chemotherapeutic regimens continue to pose a threat to fertility. The only fertility preservation methods available to pre-pubertal children of both genders is cryopreservation of gonadal tissue, a highly experimental intervention, or shielding/re-location of reproductive tissue in the setting of radiation. These techniques are available in the post pubertal population as well, but post pubertal patients also have the option for cryopreservation of gametes, a process that is much simpler in males than females. For this reason, prior to the initiation of therapy, sperm banking should be considered standard of care for males, while consideration of embryo or oocyte cryopreservation should be limited to those females at risk of developing ovarian failure. Attention to reproductive health and fertility preservation should continue after the completion of therapy. Establishing programs that streamline access to current fertility preservation techniques will assist in ensuring that all eligible patients can avail themselves of current options.

## 1. Introduction

The refinement of therapeutic interventions and supportive care has resulted in cure rates upwards of 80% in children and adolescents diagnosed with cancer, with subsequent estimates that 1 in 530 young adults between the ages of 20 and 39 is a survivor of a childhood cancer [1]. It has long been recognized that toxicities from cancer directed therapy arising during treatment could be permanent and that new complications could manifest well into the future [2]. These complications include damage to the reproductive system in both males and females resulting in impaired fertility [3,4].

Newly diagnosed patients, survivors of childhood and adolescent cancer, and their families have identified that the retention of fertility is an important aspect of their lives [5,6], and note high levels of distress with loss of fertility [7]. Advances in the field of reproductive endocrinology have increasingly offered the opportunity for the preservation of fertility in those at risk [8]. Multiple organizations internationally have recommended that, at a minimum, all individuals of reproductive age receive information about fertility risk, options for fertility preservation and referrals to the appropriate health care providers [9,10,11,12,13,14,15,16]. Yet, putting these recommendations into practice has remained a challenge. Despite the establishment of the field of oncofertility, a recent survey that compared recollections of discussions of fertility at diagnosis found static rates of discussion in 2004 and 2011, 60% *vs.* 57% respectively for males and 50% *vs.* 45% for females. In addition, while males who had discussions about fertility were satisfied with the content of those discussions, females were not [17].

Studies of health care professionals have also suggested that risks for infertility are not routinely discussed [18]. Reasons include lack of knowledge or training on the subject, language/cultural barriers between the patient and the physician, the perception that fertility preservation discussions add stress to cancer patients’ situation, general uncertainty about the success of fertility preservation methods or patient ability to afford fertility preservation, concerns about patients with a poor prognosis or late stage disease not being appropriate candidates for fertility preservation [19], concerns about information “overload” [20] and concerns about delaying the start of treatment [21]. Similar to the patient experience described above, practitioners surveyed have described that they are more likely to discuss and refer a male patient for fertility preservation than a female patient [21].

Providing fertility preservation support in this population requires prior knowledge of who is at risk and what options are available and having an infrastructure in place for patient counseling and referral. Institutional programs that systematically address this issue have begun to be implemented [22] in an effort to meet the recommendations of the guidelines cited above. This article will summarize risks for infertility and options for preservation in male and female patients diagnosed with a childhood cancer.

## 2. Fertility Preservation in Females

### 2.1. Risk for Infertility

Paramount in importance when considering infertility risks in females is that their full reserve of oocytes are present at birth and are depleted over time. At the onset of puberty, approximately 400,000 follicles remain within the ovaries. Through menstruation, atresia and apoptosis a progressive decline in ovarian primordial follicles continues during a healthy woman’s reproductive lifespan. Since fertility is related to the number of oocytes present at a given time, fertility begins to wane when a woman reaches her late 20s, drops more significantly in the mid to late 30’s and is no longer a possibility following menopause [23].

Cancer directed treatment can cause depletion of the existing oocyte reserve. Alkylating agents, particularly cyclophosphamide and procarbazine [24], and heavy metals have been found to be the chemotherapeutic agents most highly toxic to the ovary. Higher cumulative doses and older age at treatment are both associated with greater toxicity [25]. The effect of age is presumably related to the smaller number of follicles in the ovaries of older patients at the time of treatment. Direct irradiation of the ovaries, by pelvic or spinal irradiation, in the 1–2 Gy dose range, can have permanent effects by causing the depletion of follicles, even in young girls [26]. The Children’s Oncology Group (COG) long term follow-up guidelines include exposure to higher cumulative doses or combinations of alkylators, specifically busulfan >600mg/m^2^, cyclophosphamide >7.5 g/m^2^, or conditioning for transplant, prepubertal gonadal irradiation of ≥10 Gy, pubertal gonadal irradiation of ≥ 5 Gy, and alkylators in combination with irradiation of the abdomen/pelvis, lumbar or sacral spine, or total body irradiation (TBI) as risk factors for hypogonadism [27]. There is some emerging evidence that ovarian reserve may already be diminished at diagnosis [28].

The degree to which treatment depletes the ovarian reserve contributes to the length of the reproductive window that remains for a given patient. If the treatment related depletion approaches the threshold for menopause this is referred to as acute ovarian failure (AOF). If the reserve is diminished, but not exhausted from treatment, then the individual may remain fertile following therapy but have an overall shortened reproductive lifespan, referred to as premature menopause (PM). With the exception of patients who receive very high dose chemotherapy as part of conditioning regimens for stem cell transplants, particularly in conjunction with radiation, young female cancer patients generally have a greater risk of developing PM than AOF. The Childhood Cancer Survivor Study (CCSS) identified AOF, assessed as self-reported amenorrhea, in 6.3% of survivors surveyed, the majority of whom had received abdominal or pelvic radiation [29]. In the CCSS non-surgical PM was 13 times higher among survivors than sibling controls. Risk factors included attained age, exposure to increasing doses of alkylating agents, radiation to the ovaries and a diagnosis of Hodgkin Lymphoma [30].

Radiation therapy may also impact fertility by causing damage to the uterus thereby limiting the ability of a survivor to carry a pregnancy to term. Lower birth weight [4], stillbirth and neonatal death [31] have been reported to occur more frequently in women previously treated with pelvic irradiation. Hysterectomy or oophorectomy has obvious implications for preservation of fertility. Doses of cranial radiation of 35–40 Gy or greater can cause hypogonadism by impacting the hypothalamus and pituitary [32]. Doses of radiation in the range of 22–27 Gy directly to the hypothalamus/pituitary have resulted in decreased hazard ratio for pregnancy in the CCSS cohort [33].

### 2.2. Options for Fertility Cryopreservation

#### 2.2.1. Oocyte and Embryo Cryopreservation

Embryo and oocyte cryopreservation are both considered non-experimental options for fertility preservation in post pubertal females, with oocyte cryopreservation having achieved this designation by the ASRM in 2012 [34]. This change in status removes what had previously been a barrier for fertility preservation in adolescents as there is no requirement for partner or donor sperm. Both interventions involve controlled ovarian stimulation of the ovaries to produce multiple mature oocytes. Oocytes are subsequently retrieved transvaginally in a procedure that, while minimally invasive, requires sedation or anesthesia. In oocyte cryopreservation the oocytes are cryopreserved following their extraction. In embryo cryopreservation the mature follicles are fertilized *in vitro* with partner or donor sperm and then cryopreserved. When fertility is desired in the future the oocytes or embryos are thawed. Embryos are placed through the cervix into the uterus, either of the patient themselves or of a gestational surrogate, if the patient’s uterus is compromised. IVF with frozen embryos has been widely studied and evaluated in the general population; live birth rates per embryo transferred are estimated at 30%–40% [9], rates specific to individual clinics can be found for example at the Society for Assisted Reproductive Technologies [35] in the United States, and the Human Fertility and Embryology Authority [36] in the United Kingdom. Oocytes are fertilized with partner or donor sperm and then implanted. Nine hundred live births using frozen oocytes have been reported as of 2009 with no increase in congenital malformations [37]. Improvements in the freeze/thaw process have resulted in success rates only slightly lower than with embryos [9]. Data on the success of these procedures specifically in adolescent cancer patients is scarce. The disposition of unutilized cryopreserved oocytes, either in the setting of excess sample, lack of need, or patient death may present less of an ethical or emotional concern to patients and families compared to embryo cryopreservation.

While embryo and oocyte cryopreservation is technically possible as a means of preserving fertility for post pubertal females, a number of practical limitations exist. Of concern to both patients and practitioners is the need to delay the start of chemotherapy to allow for controlled ovarian stimulation of the ovaries. Recently protocols have begun to be developed where stimulation begins in the luteal (second half) of the menstrual cycle [38] and may provide a more flexible schedule for the utilization of hormonal stimulation. However, depending on the urgency to begin cancer treatment, even this time frame may not be feasible. Cost can be a barrier although this varies greatly by country. For example, in the United States oocyte and embryo cryopreservation and future IVF, are expensive with costs upwards of $10,000 [39]. Many insurance companies do not cover these costs because cancer patients do not meet the definition of infertility (namely having tried to conceive unsuccessfully for greater than one year). Resources exist, including Sharing Hope [40], administered through Livestrong, which can assist with the provision of the hormone medications and discounted rates among reproductive endocrinologists. On the other hand, in New Zealand, for example, retrieval, fertilization if desired, cryopreservation, and storage of gametes and/or embryos for up to 10 years are covered by District Health Boards [14]. Whether sufficient time and resources exist to freeze embryos or oocytes prior to the start of treatment is a decision that must be reached on an individual basis between patient, parent and medical practitioner.

#### 2.2.2. Use of Gonadotropin Releasing Hormone Analogues

The use of GnRH analogues (both agonists and antagonists) to lessen the effect of chemotherapy on follicular depletion has long held appeal as this is much less invasive compared to the cryopreservation of reproductive tissue [41]. Most early studies evaluating their efficacy have been hampered by small numbers, retrospective design and inconsistent outcome measures, making it difficult to draw definitive conclusions [42]. More recently, prospective, randomized studies have been undertaken to address this question. Three such studies in young adult women with breast cancer demonstrated decreased rates of amenorrhea in those patients who received GnRH analogues [43,44,45]; three trials in young adult patients with breast cancer [46,47] and Hodgkin lymphoma [48] demonstrated no protection of ovarian reserve using GnRH analogues. Two of the studies were stopped prematurely when interim analyses demonstrated futility [47,48]. The 2013 ASCO guidelines recommend that patients be informed that there is insufficient evidence showing that ovarian suppression via the use of GnRH analogues protects fertility and should not be relied upon for this indication [11]. Most recently, the POEMS trial randomized breast cancer patients to either standardized chemotherapy or standardized chemotherapy plus goserelin [49]. That trial demonstrated a significant reduction in ovarian failure defined as amenorrhea of six months plus elevated FSH at two years. They also demonstrated a significantly increased odds ratio (OR = 2.22, 95% CI: 1.00–4.92, p = 0.05) for pregnancy in the experimental arm. This is the first study to demonstrate a difference in pregnancy rate. The impact of this study on future guideline recommendations remain to be seen.

#### 2.2.3. Ovarian Tissue Cryopreservation

Ovarian tissue cryopreservation involves surgically removing all or a part of the cortex of the ovary, which contains thousands of primordial follicles. The resected tissue is cut into strips and cryopreserved. Because the process does not require hormonal stimulation it is the only fertility preservation technique that is available to pre-pubertal girls or females in whom initiation of treatment cannot be delayed [50,51]. Following completion of treatment the ovarian tissue can be thawed and transplanted orthotopically, *i.e.*, at the site of the ovaries, or heterotopically, *i.e.*, at another location. Once transplanted the follicles within the ovary have the potential to mature when appropriately stimulated. Approximately 30 live births have been reported utilizing this procedure of removal and orthotopic re-transplantation in individuals who were post-pubertal at the time of retrieval [52]. No births have been reported in individuals who were pre-pubescent at the time of tissue cryopreservation.

At this time ovarian tissue cryopreservation remains highly experimental without reliable means of ensuring maturation of the follicles and as such should be offered within the context of an IRB approved protocol. In addition, there are a number of challenges that exist. Obtaining tissue requires a surgical procedure with anesthesia, although ideally this could be coordinated with other procedures required for evaluation or treatment [53]. A significant area of current research focuses on attempting to mature the follicles *in vitro*. The ability to reliably do this would allow much greater control of the maturation process and precludes the need to transplant tissue back into the body [54]. This is a critical goal as the return of ovarian tissue into the body may in fact re-introduce cancerous cells, particularly in hematologic malignancies [55].

#### 2.2.4. Protection of Ovarian Function

Oophoropexy, the relocation of the ovaries outside of the radiation field, may mitigate ovarian damage, although radiation scatter can still cause follicle depletion [56]. Shielding of the ovaries during radiation therapy should be considered standard of care, when ovaries are not in the treatment field.

#### 2.2.5. Decision Making

Making decisions about fertility preservation requires sufficient information and the time to weigh the benefits and limitations of the proposed procedures. As noted above discussions between medical teams and patients/parents are still not routine practices. Anderson *et al.* examined how frequently physicians of children and adolescents diagnosed with cancer discussed fertility preservation and how often referrals were made. Prior to therapy a discussion on risk for fertility was had with 86% of post pubertal females, fertility preserving techniques were discussed with 13% of females and 4 patients (1%) were referred to a fertility specialist [57]. Low level of referrals may reflect the barriers related to timing and cost for established methods of fertility preservation in females and/or concern about experimental procedures but a recent small, qualitative study by Peddie *et al.* offers some insight into how the physician/patient interaction may affect decision making and referrals. Fifteen medical professionals and 18 female cancer patients aged 17–45 were interviewed. Medical professionals were less likely to have detailed discussions with women than with men and took many more individual factors (age, current parental status, and likelihood of relapse) into account when deciding how much information to provide. Women felt that pursuing fertility preservation options was discouraged and that conversations strongly emphasized the risk of delaying therapy. They perceived that individual factors were being taken into account in terms of how much information they were given, and they felt that they were not given the opportunity to have an adequate discussion to help make their decision [20]. This data suggests that additional research is needed to explore the content of the discussions being had and what information patients and parents need in order to make informed decisions [58], particularly as technologies improve.

### 2.3. Considerations Post-Therapy

After therapy has been completed patients exposed to potentially gonadotoxic therapies should have their ovarian function monitored. Traditionally, assessment of diminished ovarian function has relied upon sustained cessation or irregularities in the menstrual cycle as well as persistent elevations in follicle stimulating hormone (FSH) indicating proximity or achievement of menopause, with accompanying infertility. In this setting, further evaluation by an endocrinologist is warranted, particularly as early ovarian failure may increase the risk for the development of osteoporosis and cardiovascular disease [29]. Patients who received gonadotoxic treatment when they were pre-pubertal may be at risk of delayed or absent pubertal development which also requires further evaluation and intervention.

Amenorrhea, both during and after therapy, is not uncommon in post-pubertal females. Those females who resume regular menstrual cycles following the completion of cancer directed therapy may still be at risk of developing premature menopause. Increasingly Anti-Mullerian hormone (AMH), which can be measured in the serum, has been utilized to provide earlier information about ovarian reserve [59,60]. Cross sectional studies of childhood cancer survivors compared with healthy controls have shown lower levels of AMH many years after the completion of therapy [61]. Currently, the Children’s Oncology Group recommends that survivors at risk for infertility (defined above) be screened with measurements of LH, FSH and estradiol at age 13 and then as clinically indicated [27]. Referrals to a reproductive endocrinologist should be initiated for survivors with signs of or an in interest in evaluating ovarian reserve. Unfortunately, even with the addition of AMH and ovarian imaging modalities it is not possible to estimate time to menopause and thus the remaining duration of the reproductive window in a given individual. Therefore, oocyte or embryo cryopreservation following completion of therapy may allow for preservation of fertility preservation in those individuals at risk of premature menopause but not ready to become pregnant.

It is encouraging to note that, even in the setting of diminished ovarian reserve [62] or clinical infertility [63], pregnancy is achievable in the survivor population. When needed, previously cryopreserved embryos or oocytes can be thawed, (and fertilized with partner or donor sperm in the setting of oocyte cryopreservation), and transferred into the uterus following adequate hormonal stimulation to prepare the uterus for implantation. For those survivors who are infertile, did not preserve embryos or oocytes but who want to carry a pregnancy, donor eggs with either partner sperm or donor sperm may be an option. When a survivor is not able to carry a pregnancy either because she has had a hysterectomy, has had sufficient radiation therapy to cause uterine insufficiency or requires ongoing imaging that would subject a fetus to radiation, gestational surrogacy, that is the use of the uterus of another female, offers the opportunity for a biological child [9]. Gestational surrogacy is not universally legal; it is regulated very differently in different countries, and on the state level in the United States, so awareness of laws and procedures in a given area is critical for success. When options for biologic parenthood are unavailable or undesirable, adoption remains a possibility for parenthood. Ascertaining the policies of a given adoption agency or country of adoption is necessary as there can be variable policies with regard to those who have had a history of cancer.

### 2.4. Risk for Infertility in Males

Spermatogenesis, the maturation of spermatogonia from spermatogonial stem cells (SSC), begins in puberty and then continues through a male’s lifetime, as the SSC are also self-renewing. The process, which takes approximately 74 days, is regulated by hormones secreted from the hypothalamic-pituitary-testicular axis. Therefore, unlike females, males do not face a defined reproductive window unless they sustain a very significant insult to the population of SSCs. While sperm quality may change over time, males continue to be able to have biologic children well into older adulthood [64].

As rapidly differentiating cells, spermatogonia are particularly sensitive to chemotherapy and radiation. Post pubertal males commonly experience temporary azoospermia during therapy that may persist for a number of years post therapy [65]. Since SSC cells proliferate at a slower rate than spermatogonia they are less vulnerable to effects of gonadotoxic therapy but can be affected nonetheless [66]. Permanent azoospermia therefore depends on the degree to which SSC are depleted; it is this mechanism that explains why pre-pubertal males are also at risk of permanent azoospermia [67]. Sexual function, *i.e.*, the ability to maintain an erection and ejaculate, can also be affected by chemotherapy and can contribute to the ability for males to father children through intercourse. Finally, surgical interventions that result in disruption of the anatomy or nerves supplying the reproductive organs may limit unassisted reproduction.

Alkylating agents such as cyclophosphamide, ifosfamide, procarbazine, and busulfan as well as cisplatin have been shown to be highly gonadotoxic, in a dose dependent fashion [68]. The testes are extremely sensitive to even low doses of radiation. Toxicity and time to recovery is dose dependent with higher doses leading to permanent azoospermia. Doses as low as 0.1 Gy to the seminiferous tubules can cause short term azoospermia. Longer term azoospermia with recovery after approximately 30 months can be seen in the 2–3 Gy range while doses of 6 Gy and greater can lead to permanent infertility by causing significant depletion of the SSC pool [69]. The COG long term follow-up guidelines define exposure to MOPP therapy ≥3 cycles, busulfan ≥600 mg/m^2^, cyclophosphamide ≥60 g/m^2^, multiple alkylating agents, any alkylator combined with testicular irradiation, pelvic irradiation or TBI, and radiation doses in the range of 3–6 Gy as risks for the development of oligospermia/azoospermia [70]. Prior to the onset of therapy azoospermia may be present in certain populations including patients with Hodgkin lymphoma [71] and testicular cancer [72].

### 2.5. Options for Fertility Preservation

#### 2.5.1. Sperm Banking

Preservation of fertility in post pubertal males is reliably accomplished by cryopreserving sperm prior to the onset of gonadotoxic therapy. This timing is important as there is the possibility that chromosomal damage can occur when the collected sperm has been exposed to gonadotoxic therapy [73]. The most common method to obtain sperm is through masturbation, which can be done in the in-patient or out-patient setting, or via referral to a sperm bank. Males who are greater than Tanner stage III should be physically mature enough to masturbate to ejaculation. Optimal procedures for the collection of sperm include abstinence 48 h prior to collection and the collection of multiple specimens, at least 24 h apart [74]. The semen sample is evaluated for sperm count, morphology and motility prior to cryopreservation. Depending on the volume, multiple vials of sperm can be cryopreserved. The sperm is thawed when needed for use in future assisted reproduction techniques. Sperm cryopreserved for as long as 28 years has shown to be viable when thawed [75]. It is advisable that all males Tanner Stage III or greater attempt sperm banking prior to the initiation of therapy, even if they are not receiving gonadotoxic therapy. The rationale for this is that most males undergo a period of azoospermia following the completion of therapy and the face of a relapse and more toxic therapy may be unable to sperm bank at that time [76].

Limitations to this form of cryopreservation are related primarily to an inability to masturbate, whether secondary to age, illness, discomfort [77] or cultural mores that prohibit masturbation. Emotional and practical issues may also be present that limit success. Adolescent males may be uncomfortable and embarrassed discussing masturbating. If sperm banking occurs in the clinic or hospital, it is essential that a private area be designated, and the adolescent assured that he will not be interrupted. If an adolescent will be banking at a sperm bank facility, it may be necessary to ensure that the facility itself has age appropriate space and material available [78].

Alternative approaches exist when masturbation is not possible. Electroejaculation (EEJ) involves the placement of a transrectal probe while the patient is under general anesthesia. Electrical stimulation is applied until ejaculation occurs and sperm is collected [79]. Sperm retrieval rates with EEJ average around 60% in the adolescent population [79,80]. If low sperm counts or obstructive azoospermia is present, sperm may also be acquired via microsurgical epididymal sperm aspiration, the aspiration of sperm from the epididymal tubule; or testicular sperm extraction (TESE). These procedures are invasive and require anesthesia but they can be combined with other surgical procedures. Sperm extracted from testicular tissue or the tissue itself can be processed and cryopreserved.

Ideally, the development of a relationship between an oncology program and a designated sperm bank can streamline the process for a given patient as time constraints related to starting therapy are often cited as an obstacle to sperm banking [81]. With a system in place to coordinate the requirements of sperm banking including paperwork, laboratory evaluation and finances, the process itself takes a relatively short period of time and should not delay therapy. Studies have demonstrated that sperm banking is feasible in the adolescent population [82,83].

#### 2.5.2. Testicular Tissue Cryopreservation

Sperm banking is not possible for pre-pubertal boys as they cannot yet produce mature spermatozoa. Cryopreservation of testicular tissue that contains SSC offers an experimental option for preservation of fertility in pre-pubertal boys and post-pubertal males who cannot produce a sperm sample [84]. Currently, there are no human applications for the use of thawed testicular tissue. Research is ongoing in animal models to develop maximally effective methods of freezing, thawing and transplanting this tissue [85,86]. A noteworthy shortcoming with testicular tissue transplantation even with improvements in technology is the potential to re-introduce malignant cells [87]. Research is also underway to evaluate methods to mature and expand SSCs *in vitro* so that they could be used with assisted reproduction techniques and to evaluate the acceptability, feasibility and safety of testicular tissue biopsies in young, newly diagnosed pediatric cancer patients [88].

#### 2.5.3. Protection of the Testes during Treatment

The testes should be shielded during radiation therapy to try to minimize the exposure to scatter radiation. Consideration can also be given to moving the testes out of the radiation field. At this time, there are no medications shown to protect males from gonadotoxicity [11].

#### 2.5.4. Decision Making

As stated in the introduction, males are often more satisfied with the information that they receive about fertility preservation prior to the initiation of therapy and much higher referral rates occur than for females [57]. In the small qualitative study by Peddie *et al.* referenced above both males and medical professionals recalled that fertility conversations at diagnosis generally encouraged sperm banking, even if the face of low risk for infertility, and was presented as a straight forward intervention, regardless of individual demographic factors [20]. Despite being more satisfied with the information that they are receiving about fertility preservation options prior to the start of therapy, males still underutilize sperm banking at diagnosis and cryopreserved sperm post therapy but rarely dispose of sperm [89]. Characteristics associated with sperm banking are younger age, better education, being childless, being single, having a higher quality of life, and being optimistic. The single most important reason was a desire to have children in the future [90]. Oncologists who advise patients to pursue sperm banking appear to have a role in increasing rates [91]. Deterrents to sperm banking include concerns about delaying therapy and worries about the consequences of children conceived from frozen sperm [89]. The latter in particular suggests that taking the time to understand, and possibly correct, the perceptions patients have about sperm banking is a critical component to fertility preservation discussions.

With regard to testicular tissue banking, Ginsberg *et al.* studied the factors involved in making a decision to cryopreserve testicular tissue among 74 patients, 12 years old or younger, and their parents who were approached to undergo this procedure. Fifty seven families consented and 48 boys had a biopsy performed. The researchers found that although diagnosis was a stressful time, they were able to consider the experimental nature of the procedure. Of note, those that did consent to biopsy felt less overwhelmed than those that refused. In those families where a biopsy was performed there was an expression of hopefulness for future scientific advances, a stronger desire to maintain the capacity for biologic offspring for their son and a hope to minimize distress if infertility did occur. For those that chose not to consent concern about the risk of the biopsy was cited as a reason [88].

### 2.6. Considerations Post Therapy

Predictions about which patients will become infertile following gonadotoxic therapy remains imprecise and exposed males may still retain sufficient fertility to sire a child through sexual intercourse [4]. The CCSS has reported a prevalence of infertility in 46% of male survivors compared to 17.5% in the sibling cohort [92] and a decreased hazard ratio of 0.56 (95th percent confidence interval 0.49–0.63) for fathering a pregnancy compared to sibling controls [3]. However, within the CCSS cohort 37% of males defined as infertile were still able to have biologic children. The authors suggest that this may point to episodic periods of infertility and fertility [92] . Although it would be desirable to have serum screening markers for infertility recent evidence suggests that FSH and inhibin B may not be suitable replacements for semen analysis in the evaluation of sperm viability [93]. Testosterone levels may provide information about pubertal status, hypogonadism and sexual functioning with decreased levels indicating prepubertal status, hypogonadism or possible sexual dysfunction [94].

Assisted reproduction offers options for biologic children for men with very low sperm counts (oligospermia) via a technique known as intracytoplasmic sperm injection (ICSI), the injection of a single sperm into an oocyte. ICSI greatly improves the likelihood that a small number of viable sperm retrieved pre or post therapy can be used for fertilization. In survivors, banked sperm can be thawed for use with *in vitro* fertilization (IVF) with or without ICSI. Pregnancy rates using this methodology are similar to those in the general population [95], and the incidence of congenital anomalies is also not significantly different from those not treated for cancer [96,97]. Yet, despite the opportunities that assisted reproduction offers males, they often do not follow up with assessments of their fertility or use of frozen sperm. It is not clear whether this is due to having sired a child via sexual intercourse, distress about confirming they are infertile, not receiving information about follow-up post treatment or not comprehending the full implication of sperm banking given at diagnosis [98]. When viable sperm is truly unavailable, male survivors can also consider using donor sperm for fertilization of a partner’s oocytes. As with females, adoption is also an option for creating a family.

**Figure 1:**
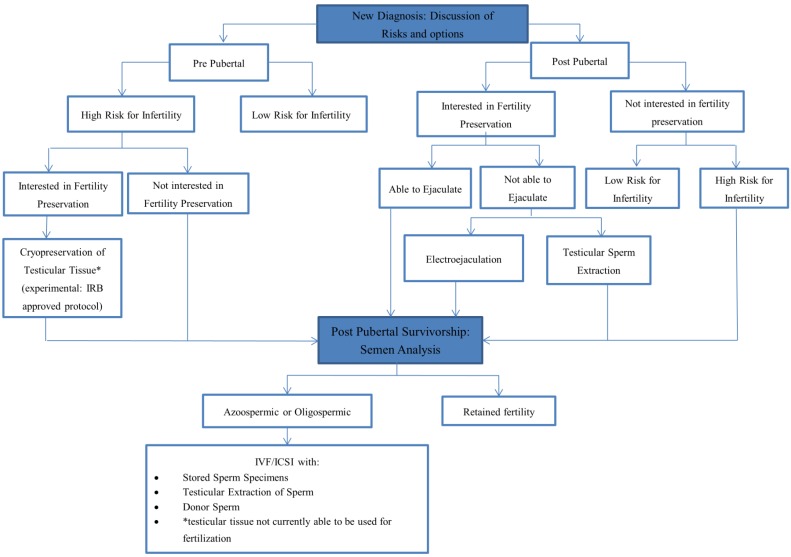
Algorithm of fertility preservation options for males.

**Figure 2:**
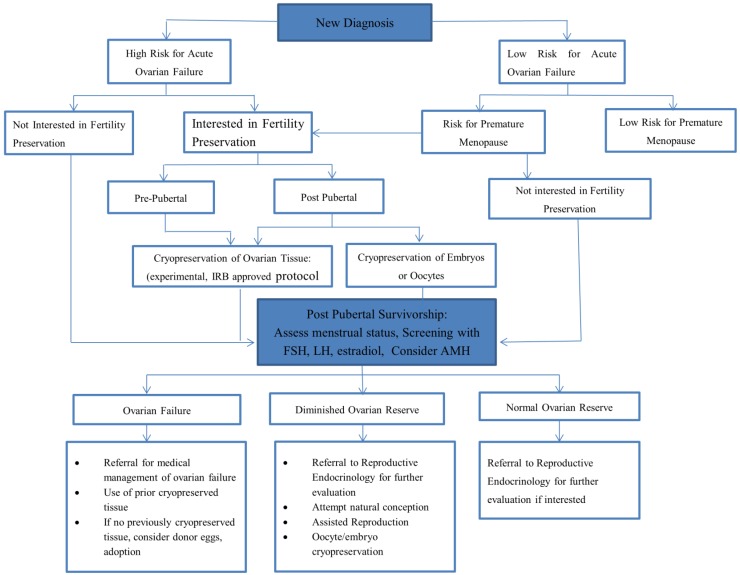
Algorithm for fertility preservation options in females.

## 3. Conclusions

With excellent survival rates and advances in the field of reproductive endocrinology, discussing risks for infertility and fertility preservation options with pediatric and adolescent patients diagnosed with cancer at diagnosis is an important component of comprehensive care. Even though such discussions are limited by inexact estimates of risks and reproductive technologies, early discussions allow patients and families to engage in decisions and may decrease the potential for regret later on. Discussion of fertility preservation should not be limited to diagnosis; survivorship is also an important time for patients to be aware of their fertility status and choices. For girls it is particularly important to monitor ovarian reserve as they have the potential to avail themselves of fertility preservation interventions post therapy. Algorithms for consideration of fertility preservation practices are provided for females and males in Figure 1 and Figure 2 respectively.

Despite an acknowledgement that discussions of fertility are important they do not occur routinely, yet these discussions are vital to help patients and families make decisions about fertility preservation. Understanding the risks for gonadal toxicity and options for fertility preservation are only the first steps to improving the likelihood that these discussions take place. Developing routine institutional practices and infrastructure is necessary given the time constraints and multiple stressors that exist at the time of diagnosis. The type of program that suits an individual institution may vary depending on the size and complexity of the practice but needs to include a mechanism that triggers identification of the target population, provides patients and families with information and facilitates access to services. The ability to consistently provide patients and families with up to date information in a timely fashion is likely to be beyond the scope of an individual physician. Identifying or creating reliable and relevant supplemental information, or directing patients to cancer focused websites is critical as information available by a patient initiated search may be inadequate and confusing especially at the time of a new diagnosis [99]. Development of fertility preservation programs, partnerships between pediatric oncology and reproductive endocrinology programs, and use of electronic medical records can help stream line the process of discussing and referring patients for fertility preservation [100,101]. These measures should be in place throughout the survivorship lifespan, from diagnosis to long term follow-up.
